# Prophylactic Inhaled Antibiotics for Ventilator-Associated Pneumonia: A Systematic Review and Meta-Analysis of Incidence and Mortality Outcomes

**DOI:** 10.1007/s00408-025-00827-1

**Published:** 2025-07-05

**Authors:** Carlos Valladares, Bryan Gregory, Sheilabi Seeburun, Ahmed Dawood Al Mahrizi, Shreya Shambhavi, Adam Kaplan, Wajahat Khan

**Affiliations:** 1 Department of Internal Medicine, Rutgers Health—RWJ Barnabas Health, Community Medical Center, 99 NJ-37, Toms River, NJ 08755 USA; 2 Department of Critical Care Medicine, Rutgers Health—RWJ Barnabas Health, Community Medical Center, Toms River, NJ 08755 USA; 3 Department of Internal Medicine, University of Malta Medical School, Msida, Malta

**Keywords:** Inhaled antibiotics, Ventilator-associated pneumonia, Critically Ill patients, Pneumonia, Mechanical ventilation

## Abstract

**Purpose:**

Ventilator-associated pneumonia (VAP) is a common ICU complication linked to high morbidity and mortality. Inhaled antibiotics may offer targeted prophylaxis, but their effectiveness has shown mixed results. This study aims to further evaluate whether inhaled antibiotics reduce VAP incidence and ICU mortality through a systematic review and meta-analysis of the most updated available evidence.

**Methods:**

A systematic review was conducted following PRISMA 2020 guidelines. Multiple databases were searched for studies published up to November 14, 2024. Ten studies including 2876 patients (1485 intervention; 1391 control) met inclusion criteria. A random-effects meta-analysis was performed to estimate pooled risk ratios (RR) for VAP incidence and ICU mortality. Risk of bias was assessed using ROBINS-I and certainty of evidence via GRADE.

**Results:**

Inhaled antibiotics significantly reduced the incidence of VAP compared to controls (RR = 0.67; 95% CI: 0.58–0.77; *p* < 0.001), but showed no significant effect on ICU mortality (RR = 0.92; 95% CI: 0.79–1.06; *p* = 0.25). Moderate heterogeneity was observed in VAP outcomes (*I*^2^ = 46.8%), while mortality analysis showed no heterogeneity. Funnel plot analysis suggested minimal publication bias, and GRADE rated the evidence as moderate in certainty.

**Conclusion:**

Inhaled antibiotics significantly reduce VAP incidence but show no clear mortality benefit. While promising for prevention, their survival impact remains uncertain. Clinical use should consider patient context and microbial patterns for targeted approach. Future research should identify high-risk subgroups, assess long-term outcomes, and evaluate antibiotic resistance.

**Supplementary Information:**

The online version contains supplementary material available at 10.1007/s00408-025-00827-1.

## Background

Ventilator-associated pneumonia (VAP) remains a significant complication in critically ill patients receiving invasive mechanical ventilation (MV), contributing to increased morbidity, prolonged ICU stays, and substantial healthcare costs [1–3]. VAP incidence varies widely, ranging from 2 to 30 cases per 1000 ventilator days, depending on diagnostic criteria and clinical settings [4–6]. Despite advancements in infection control protocols, including oral hygiene, subglottic secretion drainage, and ventilator weaning strategies, VAP continues to pose a major challenge due to microaspiration, biofilm formation on endotracheal tubes, and limitations in systemic antibiotic penetration [1, 7–10]. VAP is also associated with increased mortality, with reported ICU mortality rates ranging from 20 to 50%, of which a significant proportion is directly attributed to the infection [11, 12].

In recent years, inhaled antibiotics have emerged as a potential prophylactic strategy for VAP prevention [13]. But does their use effectively reduce the incidence of VAP and improve ICU outcomes? Unlike systemic antibiotics, inhaled agents achieve higher drug levels in tracheal secretions, surpassing minimum inhibitory concentration (MIC) breakpoints for common pathogens [14]. Several delivery methods, including jet, ultrasonic, and vibrating mesh nebulizers, have been explored to optimize drug deposition in the lungs, with vibrating mesh nebulizers being the most effective [15].

Despite their potential, the effectiveness of inhaled antibiotics in preventing VAP remains uncertain, as previous studies have reported mixed results due to variations in study designs, patient populations, and methodological inconsistencies [16, 17]. Given the rising prevalence of multidrug-resistant (MDR) bacteria in ICU settings, the role of inhaled antibiotic prophylaxis warrants further evaluation [3]. Additionally, while inhaled antibiotics may reduce VAP incidence, their impact on ICU mortality remains unclear, requiring further investigation.

This review updates the evidence base by incorporating recently published trials—notably the large multicenter RCT by Ehrmann et al. (2023)—and highlights study-level variation by antibiotic class and delivery method, enhancing the clinical relevance of its findings.

This systematic review and meta-analysis aim to provide updated, comprehensive evidence on the efficacy of inhaled antibiotics in preventing VAP among mechanically ventilated patients. Additionally, it seeks to evaluate their impact on ICU mortality to determine whether reducing VAP incidence translates to a survival benefit and to help clarify the role of inhaled antibiotics in VAP prophylaxis for future clinical guidelines.

## Methods

A systematic review and meta-analysis was performed utilizing the Preferred Reporting Items for Systematic Reviews and Meta-Analysis (PRISMA 2020) guidelines [18]. All major relevant databases (PubMed, Web of Science, Embase, Scopus, Cochrane), as well as large gray literature sites (Google Scholar and World Cat), were searched for articles on inhaled antibiotics and VAP prevention published before November 14, 2024. Manuscripts were identified using these search phrases: (“Nebulized” OR “Inhaled” OR “Nebulizer” OR “Vaporizer” OR “Inhalators” OR “Atomizer” OR “Aerosol” OR “Inspiration” OR “Respiratory” OR “Volatization”) AND (“prevention” OR “prophylaxis” OR “Preventive”) AND (“VAP” OR “Ventilator Associated Pneumonia” OR “Ventilator-Associated Pneumonia”). After this search 4491 studies were identified, however after titles and abstract were exported to Rayyan.ai 1994 duplicates were found [19].

### Inclusion and Exclusion Criteria

**Fig. 1:**
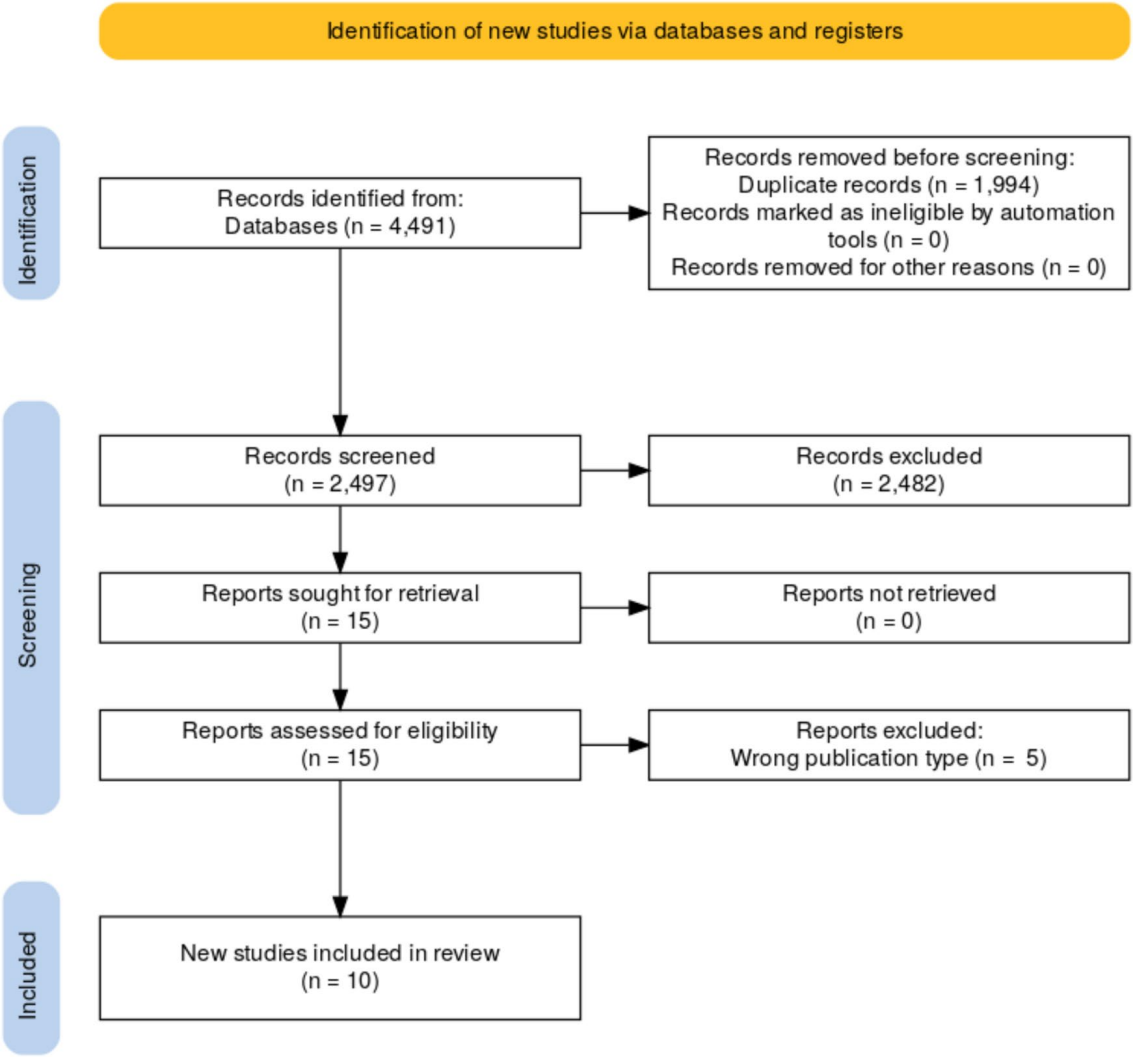
PRISMA 2020 flow diagram of study selection process

Full-text manuscripts that evaluated inhalation of antibiotics in the prevention of VAP were included. Prophylaxis was defined as the administration of inhaled antibiotics prior to the onset of clinical signs of VAP, with the intent to prevent infection. Studies were included based on the prior mentioned prophylactic effect. Studies administering inhaled antibiotics for early or presumptive treatment were excluded. Manuscripts that evaluated inhalation of antibiotics for treatment of VAP or who did not include antibiotics but instead compared other inhaled substances for VAP prevention were excluded (*n* = 2482) (Fig. 1). In addition, studies in which the full text was not available were excluded (*n* = 0). Using these criteria, titles and abstract were screened independently by two authors and in case of disagreements a third author would be called to settle. Of the eligible 15 articles, four [20–23] were excluded due to being the wrong publication type and one [24] was excluded for not having a control group. Ten were appraised fully. Finally, a total of ten studies were included after inclusion and exclusion criteria were applied (Table 1) [25–34].

**Table 1:** Summary of included studies

Study (year)	Type of trial	Number of patients	ICU population	Antibiotic intervention (dose)	Antibiotic class	Control (placebo used)	Mode of administration
Karvouniaris et al. [25]	RCT	84	Medical ICU	Colistin (2 MIU q8h)	Polymyxin	Normal Saline	Nebulized
Klastersky et al. [26]	RCT	42	Medical ICU	Gentamicin (80 mg q12h)	Amino-glycoside	Normal saline	Endotracheal
Klick et al. [27]	RCT	744	Surgical ICU	Polymyxin B (2.5 mg/kg/day)	Polymyxin	Dextrose 5% in water	Nebulized
Lode et al. [28]	RCT	77	Mixed (medical/surgical)	Gentamicin (160 mg q8h)	Amino-glycoside	Sterile water	Endotracheal
Wood et al. [29]	RCT	20	Trauma ICU	Ceftazidime (15 mg/kg/12 h)	3rd generation cephalosporin	Normal saline	Nebulized
Claridge et al. [30]	Non-RCT	52	Trauma ICU	Cefepime (2 g q8h)	4th Generation Cephalosporin	Standard care	Nebulized
Ehrmann et al. [31]	RCT	860	Medical ICU	Amikacin (400 mg q12h)	Amino-glycoside	Normal saline	Nebulized
Greenfield et al. [32]	RCT	25	Medical ICU	Gentamicin (40 mg q8h)	Amino-glycoside	Normal saline	Endotracheal
Rouby et al. [33]	Non-RCT	251	Surgical ICU	Tobramycin (300 mg q12h)	Amino-glycoside	Sterile water	Nebulized
Rathgeber et al. [34]	RCT	40	Mixed (medical/surgical)	Gentamicin (80 mg q12h)	Amino-glycoside	Normal saline	Endotracheal

**RCT:** randomized control trial; **ICU:** intensive care unit

ICU population type is based on study-reported patient demographics and clinical setting

### Data Collection and Analysis

Aside from each study's descriptive statistics, incidence and mortality data were extracted. In addition, information on antibiotic class, delivery method, and ICU population type (e.g., trauma, surgical, medical) was extracted where available. Definitions of ventilator-associated pneumonia (VAP) varied across studies. In most cases, diagnosis appeared to be based on clinical criteria per institutional practice, although explicit definitions were often not reported. For VAP prevention, the ‘event’ was defined as development of VAP and ‘no-event’, defined as ‘prevention of VAP’. For mortality, the ‘event’ was defined as death, while ‘no-event’ was defined as survival. Statistical analysis was performed using proportional meta-analysis with a random-effects approach using RStudio (Version 2024.12.0 + 467) [35]. A proportional meta-analysis using a random-effects model was performed to estimate pooled risk ratios (RR) for VAP prevention and ICU mortality. A random-effects model was used for both VAP incidence and ICU mortality to account for potential between-study variability. Publication bias was assessed using funnel plots and Egger’s test.

Heterogeneity of the study results was assessed primarily through Q-statistics and the *I*^2^ ratio (*I*^2^ = *τ*^2^/*H*^2^). If the *p*-value of the *Q*-statistic in the homogeneity test is not significant, then the null hypothesis cannot be rejected. Taking the alternative hypothesis would mean that the variation between treatment results is not likely due to random chance alone, but to an unknown external variable that biases results. In contrast, a significant *p*-value indicates heterogeneous group effect sizes. Lower *I*^2^ indicates fewer differences between studies, with observed differences mostly resulting from differences in treatment effects, rather than bias from another variable (i.e., study design).

Tau-squared is an estimate of the absolute variation between effect sizes, ignoring random chance alone. Random-effects models enable analysis of variance higher than what is expected by chance alone, distinguishing influence of external variables from treatment effect, while a less conservative common effects model does not. To analyze variance, H2 ratios were also reported with a value of 1 representing equivalent variance between both models. In this case, low heterogeneity can be explained by no variation in study effect sizes that exceeds what is expected from random chance.

### Risk of Bias and Certainty of Evidence Assessment

Bias was independently assessed based on their respective study design by two authors using the following tools. Randomized control trials and non-randomized trials were evaluated using ROBINS-1. Data were reported graphically using the ROBVIZ stoplight plot and summary chart creation tool [36]. Manuscripts were also categorized by study design to evaluate for methodological quality using the Grading of Recommendations Assessment, Development and Evaluation (GRADE) method [37]. Disagreements between the two author's decisions were to be settled by a third author, but this was not needed.

## Results

A total of 10 studies were included in the meta-analysis, with a combined total of 2876 patients: 1485 in the intervention group (inhaled antibiotics) and 1391 in the control group. These studies provided data on binary outcomes for VAP prevention (success: no VAP, failure: occurrence of VAP) and ICU mortality (event: death, no event: survival) for both treatment and control groups.

### Effect of Intervention

**Fig. 2:**
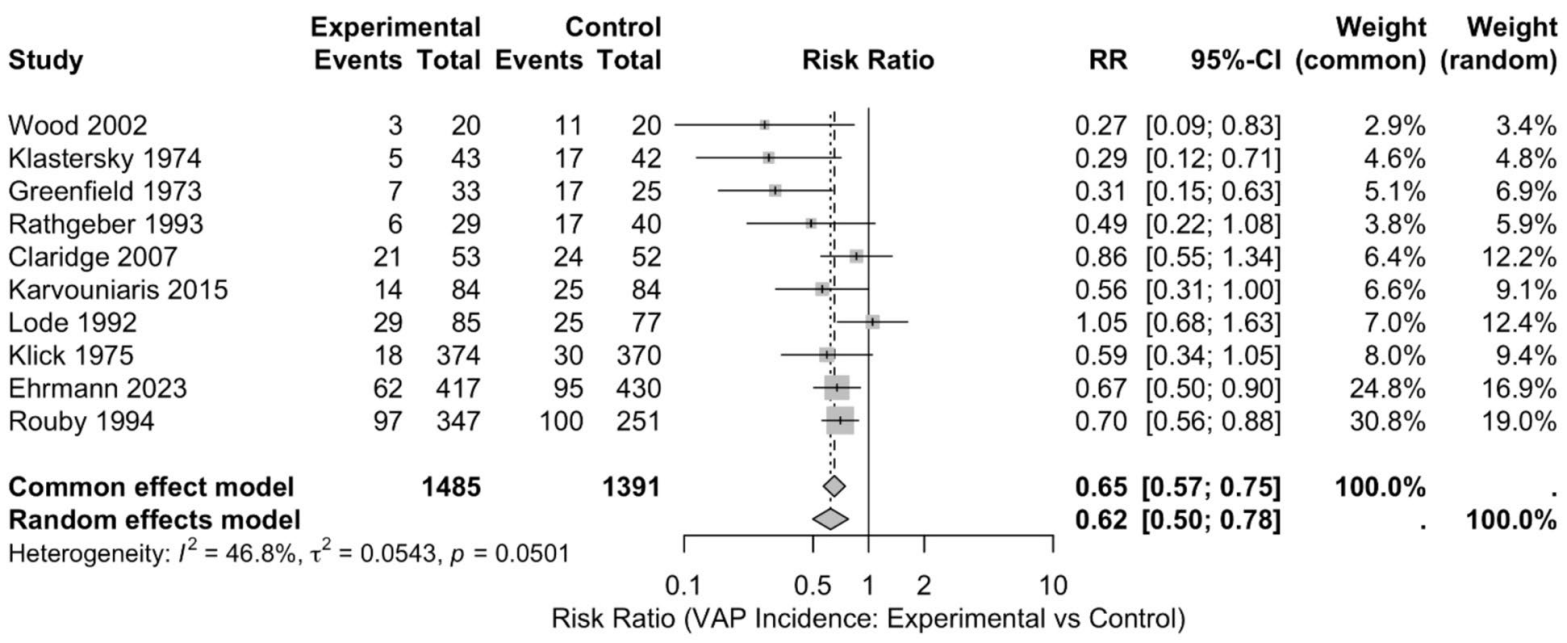
Forest plot showing the effect of inhaled antibiotics on ventilator-associated pneumonia (VAP) incidence

Overall, the use of inhaled antibiotics for the prevention of ventilator-associated pneumonia (VAP) showed a significant reduction in the incidence of VAP. The pooled risk ratio (RR) using a random-effects model was 0.67 (95% CI: 0.58–0.77), with a significant p-value of 0.0001, indicating a statistically significant reduction in the risk of VAP among patients receiving inhaled antibiotics when compared to controls (Fig. 2).

Individual study risk ratios varied, with the study by Rouby et al. showing the most substantial effect size RR = 0.70 (95% CI: 0.56—0.88). Conversely, the study by Lode et al. reported a risk ratio of 1.05 (95% CI: 0.68—1.63), indicating no significant effect. The study performed by Rouby et al. contributed the most weight (36.4%) to the pooled estimate, followed by Ehrmann et al. (22.3%), due to their large sample sizes and lower variance. While no formal subgroup meta-analyses were performed, descriptive variation was noted across studies. Polymyxins such as colistin and polymyxin B were typically used in ICUs with high prevalence of multidrug-resistant organisms. Aminoglycosides were the most frequently used antibiotic class overall, while cephalosporins such as ceftazidime and cefepime were predominantly employed in trauma-focused ICUs.

**Fig. 3:**
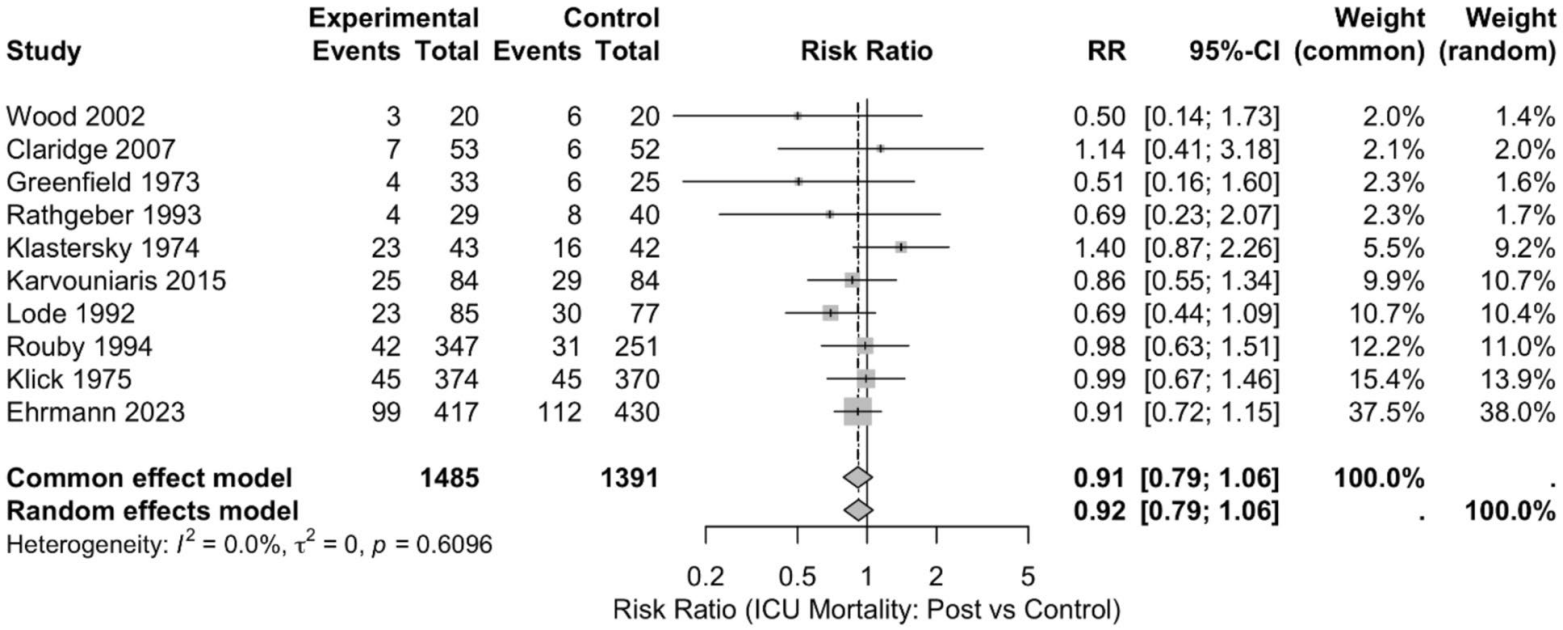
Forest plot of the effect of inhaled antibiotics on ICU mortality

As for mortality, the RR was 0.92 (95% CI: 0.79; 1.06), with a p-value of 0.2456, indicating no statistically significant difference in mortality between inhaled antibiotic and control groups (Fig. 3).

The study by Karvouniaris et al. reported the largest reduction in ICU mortality RR = 0.86 (95% CI: 0.55–1.34), though this was not statistically significant. While Ehrmann et al., which had the largest weight (38%), showed no significant difference RR = 0.97 (95% CI: 0.81–1.15). Greenfield et al. and Rathgeber et al. reported the lowest weights in the meta-analysis and found no conclusive differences RR = 1.14 (95% CI: 0.41–3.18), and RR = 0.69 (95% CI: 0.23–2.07), respectively.

### Heterogeneity

The test of homogeneity for VAP incidence revealed a *Q* = 16.91 (df = 9, *p* = 0.0501). The effect sizes had a variance of *T*^2^ = < 0.0001, the ratio of variance in the fixed effect model compared to random-effects model was *H* = 1.37, and the total heterogeneity variations attributed was *I*^2^ = 46.8%, suggesting a moderate heterogeneity among the studies. For mortality, the heterogeneity (*I*^2^) was 0.0%, indicating no observed heterogeneity among the included studies.

### Trial Sequential Analysis (TSA)

TSA was conducted to evaluate whether the current cumulative evidence was sufficient to draw firm conclusions. For VAP incidence, the cumulative *z*-curve crossed the conventional significance boundary early, indicating a nominally significant benefit, but did not cross the more conservative O’Brien–Fleming monitoring boundary. This suggests that although a reduction in VAP incidence is likely, the diversity-adjusted required information size has not yet been reached, and further studies may still impact the conclusion. For ICU mortality, the cumulative *z*-curve remained within both conventional and sequential monitoring boundaries across all information fractions, providing no evidence of a significant mortality benefit. TSA plots for both VAP incidence and ICU mortality are presented in Supplementary Fig. S2 and S3, respectively.

### Leave–One–Out Analysis

Leave-one-out analysis was performed to assess the influence of individual studies on pooled outcomes. For VAP incidence, omitting any single study did not significantly alter the overall risk ratio, with estimates consistently ranging from 0.63 to 0.72. All confidence intervals remained below the null, affirming robustness of the finding. Similarly, the ICU mortality analysis showed pooled RRs ranging narrowly from 0.88 to 0.95 across omissions, with no significant change in interpretation. These findings confirm that no single study disproportionately influenced the meta-analytic conclusions. Supplementary Figures S4 and S5 illustrate the leave-one-out sensitivity plots for VAP incidence and ICU mortality.

### Funnel Plot

**Fig. 4:**
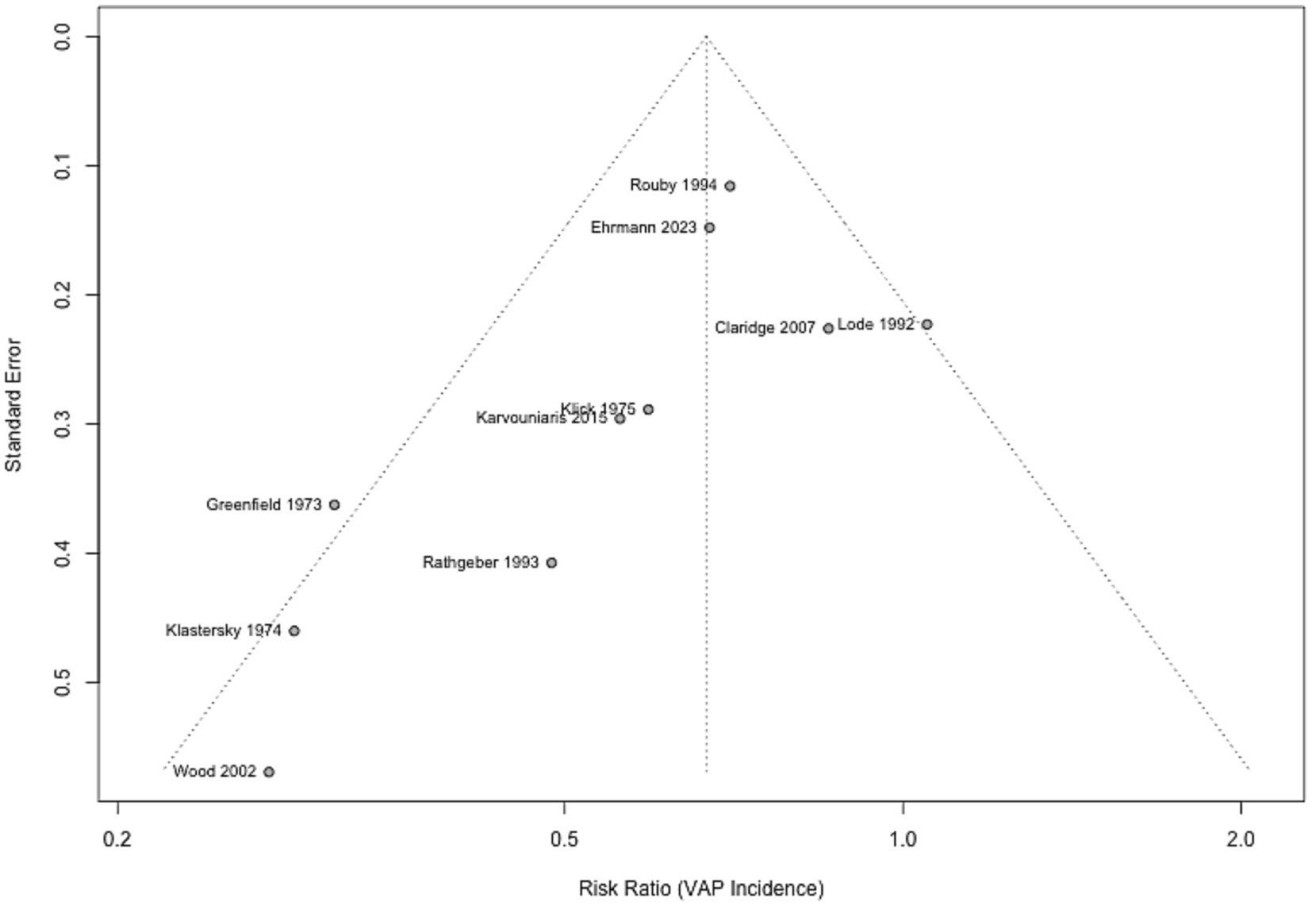
Funnel plot assessing publication bias for VAP incidence outcome

A funnel plot was generated to assess the potential for publication bias. The plot appeared mostly symmetrical, suggesting that publication bias is unlikely to significantly impact the results of this meta-analysis. However, some slight asymmetry in the lower left quadrant may indicate the presence of small study effects or potential bias (Fig. 4).

### Risk of Bias and Certainty of Evidence Assessment

**Fig. 5:**
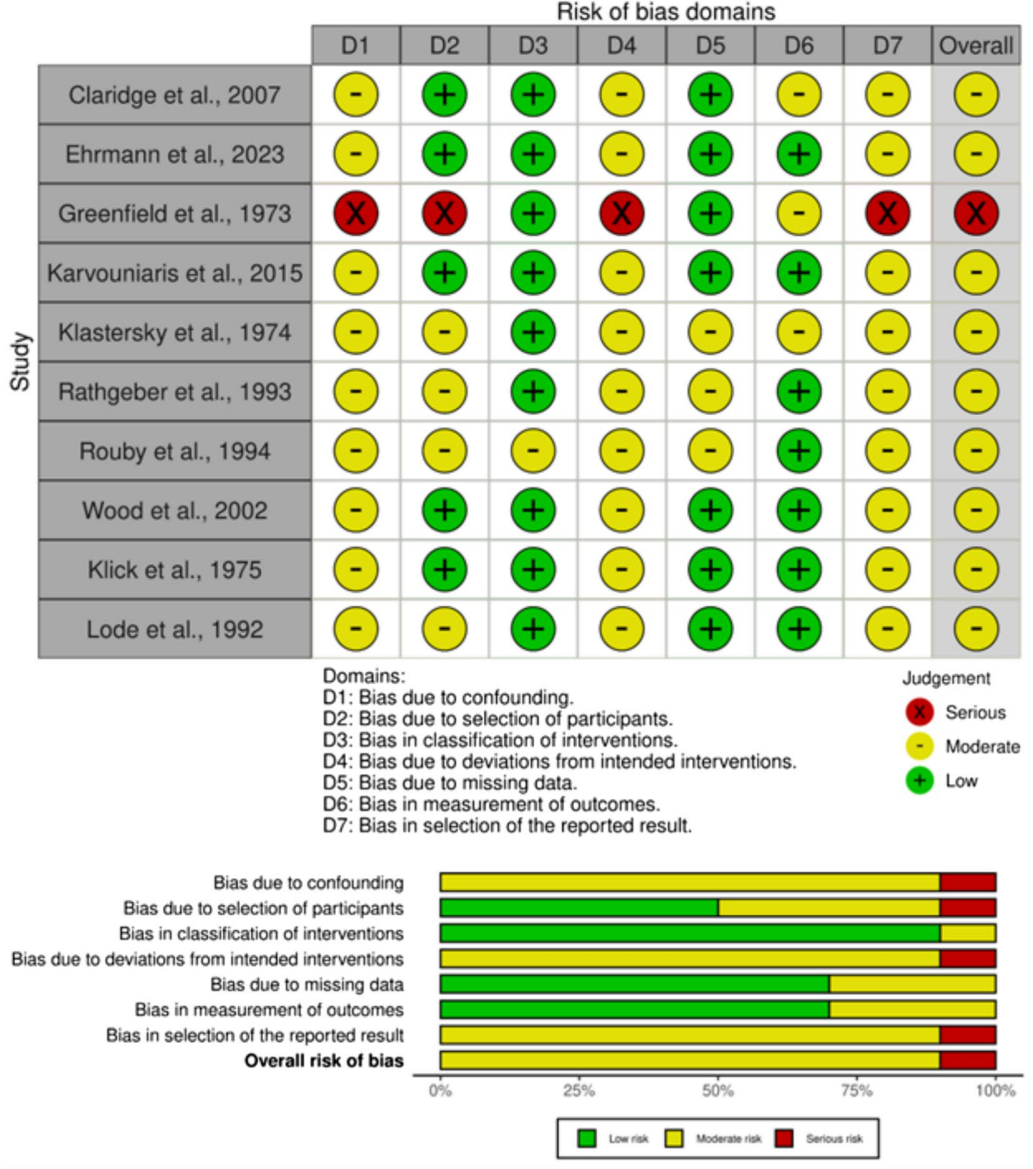
Summary of risk of bias assessments using ROBINS-I tool. Traffic light plot (top), bar plot (bottom)

Assessment of each of the ten studies was performed using Cochrane’s ROBINS-1 V2 tool [38], which revealed mainly moderate bias in each study (Fig. 5). Based on GRADE's overall analysis, the certainty of evidence was rated as moderate for most outcomes, with some domains downgraded due to study-level risk of bias or imprecision. Table S1 (Supplementary Material) summarizes the GRADE evaluation for VAP incidence and ICU mortality across all included studies.

## Discussion

Our systematic review and meta-analysis incorporated several studies that aimed to evaluate the efficacy of inhaled antibiotics in VAP prevention. The results demonstrated that the use of inhaled antibiotics significantly reduced the incidence of VAP with a 33% relative risk reduction compared to controls. However no significant difference in ICU mortality was found between groups, suggesting that while inhaled antibiotics may prevent infections, they do not confer a survival benefit in the general ICU population. The GRADE assessment rated the certainty of evidence as moderate, in part due to moderate heterogeneity, reflecting differences in patient risk profiles, diagnostic criteria, and antibiotic regimens. The GRADE assessment rated the certainty of evidence for VAP prevention as moderate, and low to moderate for ICU mortality (Table S1, Supplementary Material). The ROB assessment revealed an overall moderate risk, with some concerns related to blinding and allocation concealment. Despite these limitations, our findings consistently support inhaled antibiotics as an effective strategy for VAP prevention.

### VAP Prevention Outcomes

Ehrmann et al. conducted a large, multicenter, double-blind, randomized controlled trial evaluating the use of inhaled amikacin in mechanically ventilated patients [31]. Their findings demonstrated a significant reduction in VAP incidence in the amikacin group (15% vs. 22% in the placebo group, *p* = 0.004). Furthermore, lower rates of infection-related ventilator-associated complications were also observed, which reinforces the role of inhaled antibiotics as a preventive measure.

Karvouniaris et al. conducted a randomized controlled trial evaluating prophylactically nebulized colistin in an ICU setting with prevalent multidrug-resistant bacteria [25]. This study showed that while nebulized colistin did not significantly reduce the overall incidence of VAP, it did lower the incidence rate and decreased occurrences of Gram-negative and multidrug-resistant VAP. This finding suggests that in specific contexts, such as ICUs with high rates of multidrug resistance gram negative bacteria, nebulized colistin may be beneficial.

Similarly, Wood et al. investigated the use of aerosolized ceftazidime for VAP prevention in critically ill trauma patients [29]. Their randomized, double-blind, placebo-controlled trial demonstrated a significant reduction (73% lower) in VAP incidence among patients receiving aerosolized ceftazidime compared to placebo. The study also noted that aerosolized ceftazidime attenuated proinflammatory pulmonary response, which could have implications for patient outcomes beyond VAP prevention. In contrast, Lode et al. conducted a study on endotracheal gentamicin prophylaxis of VAP in ICU patients [28]. Their findings showed no significant reduction in VAP or mortality rates. This highlights the variability of outcomes on antibiotic choice and administration methods.

### Study Level Variability and Outlier Incidence Rates

Some studies included in this analysis, such as those by Wood, Greenfield, and Klastersky, reported VAP incidence rates that appear considerably higher than the widely cited range of 2–30 cases per 1000 ventilator days. These elevated rates may be attributed to differences in diagnostic criteria, patient severity, or study period and design. The inclusion of such outliers likely contributed to observed heterogeneity and should be taken into account when interpreting the pooled results. It is also worth noting that several studies demonstrating the most pronounced reduction in VAP incidence also reported unusually high VAP rates in their control groups. This pattern, previously observed in other reviews on VAP prevention, may exaggerate the apparent efficacy of inhaled antibiotics due to inflated baseline risk. Readers should interpret large effect sizes with caution, particularly in studies with control group VAP rates exceeding commonly reported background incidence. This reinforces the need for consistent diagnostic criteria and real-world incidence thresholds in future trials.

### Mortality Outcomes

While the primary focus of this meta-analysis was VAP prevention, our analysis also included data on ICU mortality. The pooled data revealed no significant reduction on overall ICU mortality between the intervention and control groups. Ehrmann et al. reported an ICU mortality rate of 23.7% in the inhaled antibiotic group versus 26.0% in the control group (*p* = 0.18), which indicates no statistical significance [31]. Similarly, Klick et al. and Rouby et al. also found no substantial differences in mortality rates between treatment and control groups [27, 33]. In contrast to these findings, Karvouniaris et al. reported ICU mortality rates of 7.1% versus 44% (*p* = 0.028), suggesting a potential mortality benefit in specific populations [25]. This variability in mortality outcomes may be attributed to differences in patient population, antibiotic selection, and study design. Notably, some of the included studies using colistin were conducted in settings with high MDR organism prevalence, although comprehensive resistance data were often not provided. Additionally, studies such as Ehrmann et al. contributed a large portion of statistical weight. Although inhaled antibiotics significantly reduce VAP incidence, the lack of corresponding mortality benefit suggests that other ICU related factors, such as sepsis progression, comorbidities, and overall antibiotic stewardship, may have a stronger impact on survival. Future studies should focus on identifying patient subgroups that may derive the most mortality benefit from inhaled antibiotic prophylaxis.

### Nebulizing Antibiotics in ICU Settings

While inhaled antibiotics offer pharmacokinetic advantages, their use is not without risks. Safety concerns include potential bronchospasm, airway irritation, and ventilator filter obstruction, particularly with certain formulations or delivery devices [39, 40]. Additionally, nebulizer type and technique can significantly influence lung deposition, potentially leading to under- or overdosing [41]. Proper staff training and standardized administration protocols are essential to minimize these risks. Moreover, while inhaled antibiotics may reduce VAP incidence, their indiscriminate use poses a risk of promoting antimicrobial resistance, especially in settings with already high multidrug-resistant (MDR) organism prevalence [42]. Given that most included studies did not report follow-up cultures or resistance patterns, the long-term ecological impact remains unclear—reinforcing the need for strong antibiotic stewardship. Clinicians should consider local susceptibility data, use inhaled antibiotics selectively, and ensure that administration protocols are optimized to prevent subtherapeutic dosing. Further studies are needed to evaluate the emergence of resistance and establish safe duration thresholds for prophylactic regimens.

Trial Sequential Analysis suggested that while current evidence supports a reduction in VAP incidence, the cumulative data still fall short of the required information size for robust confirmation. In contrast, the mortality signal remains indeterminate, and more data are needed before clinical guidance can be firmly based on these endpoints. Furthermore, the stability of our findings across leave-one-out analyses confirms that no single study unduly influenced pooled results, increasing the reliability of our conclusions across both VAP incidence and ICU mortality outcomes.

### Limitations

While the findings are promising, several limitations should be considered. First, heterogeneity among the studies—such as patient population, antibiotic regimen, and treatment durations—may affect the generalizability of our findings. Second, different diagnostic criteria for VAP across studies could introduce potential bias and impact reported incidence rates. Third, the limited number of high-quality randomized controlled trials restricts the strength of evidence, highlighting the need for more standardized protocols in future trials. Additionally, data on long-term outcomes, antibiotic resistance patterns, and adverse effects remains scarce and necessitates further investigation.

Although sufficient data were available to categorize studies by antibiotic class, ICU population type, and delivery method, formal subgroup meta-analyses were not performed. The relatively small number of studies per subgroup and potential for underpowered comparisons may have limited interpretability; future reviews should explore these subgroup effects using formal statistical methods. Additionally, sensitivity analysis excluding large trials such as Ehrmann et al. was not conducted but may be warranted in future reviews to assess the robustness of pooled estimates.

## Conclusion

While inhaled antibiotics show promise in reducing the incidence of VAP, their effect on mortality is less clear. The variability in study outcomes underscores the importance of considering individual patient contexts, local microbial patterns, and specific antibiotic regimens when implementing inhaled antibiotic prophylaxis in clinical practice. Future research should focus on investigating mortality as well as antibiotic resistance and long-term clinical outcomes.

## Supplementary Information

Below is the link to the electronic supplementary material.

Supplementary file1 (PDF 1370 kb)

## Data Availability

No datasets were generated or analysed during the current study.
