# Supplementary material for: Prophylactic Inhaled Antibiotics for Ventilator-Associated Pneumonia: A Systematic Review and Meta-Analysis of Incidence and Mortality Outcomes
Source: Lung. 2025 Jul 5;203(1):75. doi: 10.1007/s00408-025-00827-1 (PMC12228672; doi:10.1007/s00408-025-00827-1)
Supplement: Supplementary file 1 — Supplementary file1 (PDF 1370 kb) [file 408_2025_827_MOESM1_ESM.pdf]

Table S1. GRADE Summary of Findings Table for VAP Incidence and ICU Mortality

Study	Risk of Bias		Inconsistency		Indirectness		Imprecision		Publication Bias		Large Effect Size		Plausible Confounding		Dose-Response Gradient		Overall Certainty of Evidence	
	Incidence	Mortality	Incidence	Mortality	Incidence	Mortality	Incidence	Mortality	Incidence	Mortality	Incidence	Mortality	Incidence	Mortality	Incidence	Mortality	Incidence	Mortality
Claridge et al., 2007	Not serious (0)	Not serious (0)	Not serious (0)	Not serious (0)	Not serious (0)	Not serious (0)	Not serious (0)	Serious (-1)	Undetected (0)	Undetected (0)	No (0)	No (0)	No (0)	No (0)	No (0)	No (0)	Moderate	Moderate
Ehrmann et al., 2023	Not serious (0)	Not serious (0)	Not serious (0)	Not serious (0)	Not serious (0)	Not serious (0)	Not serious (0)	Not serious (0)	Undetected (0)	Undetected (0)	No (0)	No (0)	No (0)	No (0)	No (0)	No (0)	Moderate	Low
Greenfield et al., 1973	Serious (-1)	Not serious (0)	Serious (-1)	Not serious (0)	Not serious (0)	Not serious (0)	Serious (-1)	Serious (-1)	Undetected (0)	Undetected (0)	No (0)	No (0)	No (0)	No (0)	No (0)	No (0)	Low	Low
Karvouniaris et al., 2015	Not serious (0)	Not serious (0)	Not serious (0)	Not serious (0)	Not serious (0)	Not serious (0)	Not serious (0)	Not serious (0)	Undetected (0)	Undetected (0)	No (0)	No (0)	No (0)	No (0)	No (0)	No (0)	Moderate	Moderate
Klastersky et al., 1974	Not serious (0)	Not serious (0)	Not serious (0)	Not serious (0)	Not serious (0)	Not serious (0)	Not serious (0)	Not serious (0)	Undetected (0)	Undetected (0)	No (0)	No (0)	No (0)	No (0)	No (0)	No (0)	Moderate	Moderate
Rathgeber et al., 1993	Not serious (0)	Not serious (0)	Not serious (0)	Not serious (0)	Not serious (0)	Not serious (0)	Not serious (0)	Not serious (0)	Undetected (0)	Undetected (0)	No (0)	No (0)	No (0)	No (0)	No (0)	No (0)	Moderate	Moderate
Rouby et al., 1994	Not serious (0)	Not serious (0)	Not serious (0)	Not serious (0)	Not serious (0)	Not serious (0)	Not serious (0)	Not serious (0)	Undetected (0)	Undetected (0)	No (0)	No (0)	No (0)	No (0)	No (0)	No (0)	Moderate	Moderate
Wood et al., 2002	Not serious (0)	Not serious (0)	Not serious (0)	Not serious (0)	Not serious (0)	Not serious (0)	Not serious (0)	Not serious (0)	Undetected (0)	Undetected (0)	No (0)	No (0)	No (0)	No (0)	No (0)	No (0)	Moderate	Moderate
Klick et al., 1975	Not serious (0)	Not serious (0)	Not serious (0)	Not serious (0)	Not serious (0)	Not serious (0)	Not serious (0)	Not serious (0)	Undetected (0)	Undetected (0)	No (0)	No (0)	No (0)	No (0)	No (0)	No (0)	Moderate	Moderate
Lode et al., 1992	Not serious (0)	Not serious (0)	Not serious (0)	Not serious (0)	Not serious (0)	Not serious (0)	Not serious (0)	Not serious (0)	Undetected (0)	Undetected (0)	No (0)	No (0)	No (0)	No (0)	No (0)	No (0)	Moderate	Moderate

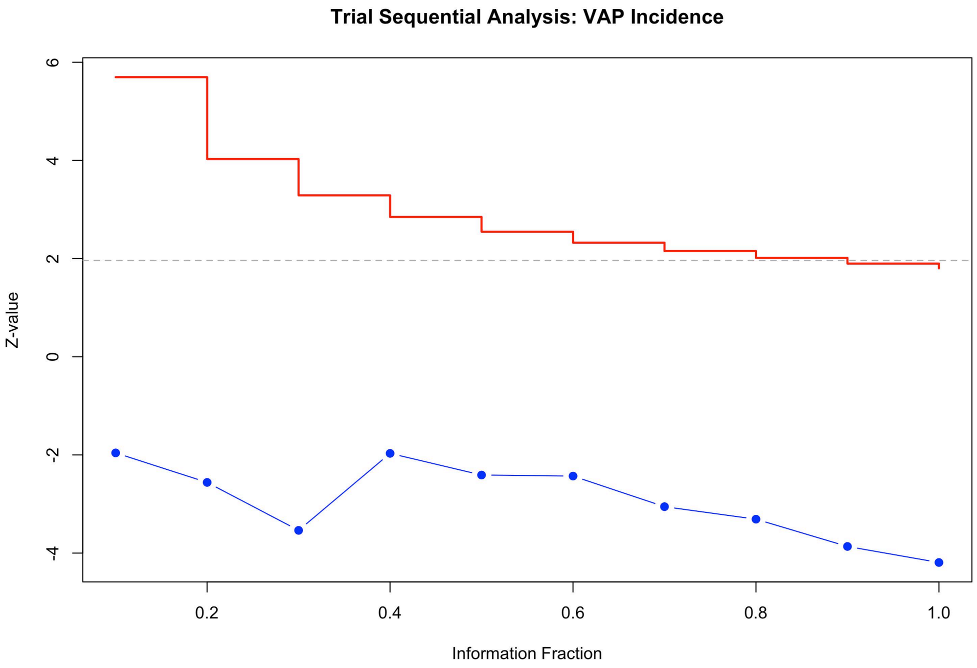

Figure S2. Trial Sequential Analysis plot for VAP incidence.

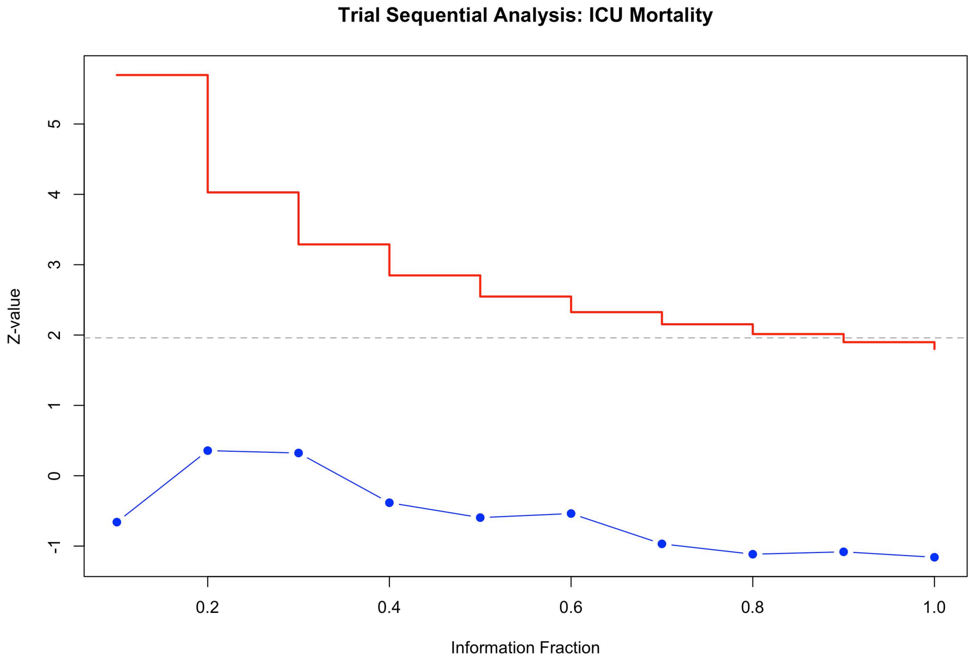

Figure S3. Trial Sequential Analysis plot for ICU mortality.

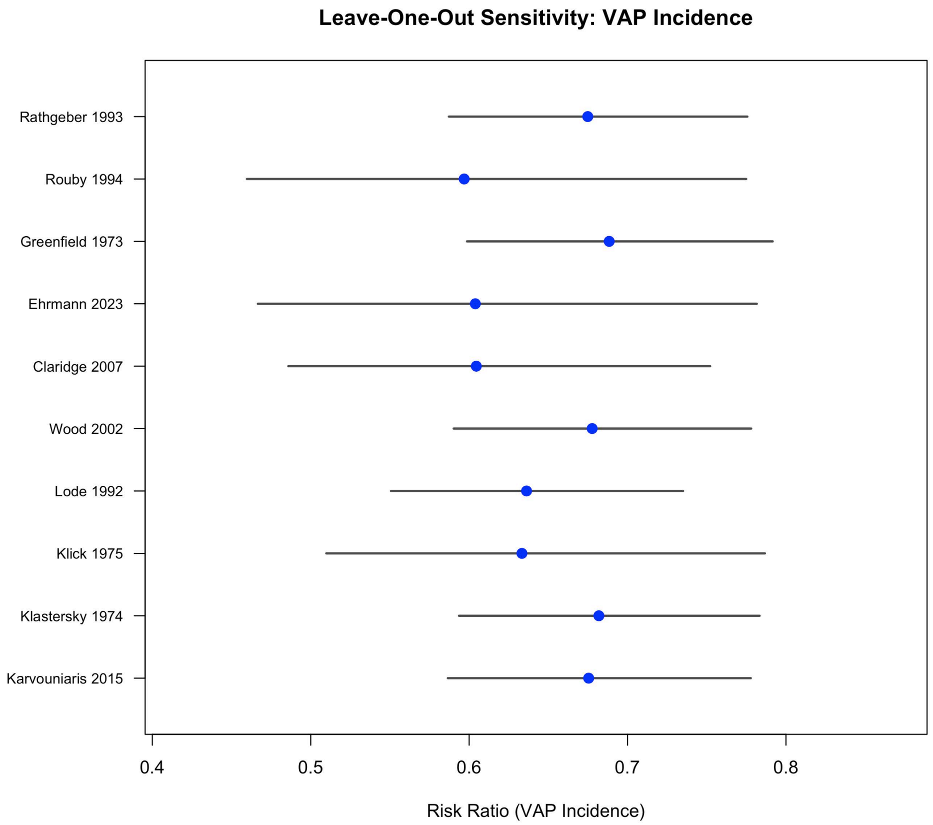

Figure S4. Leave-one-out sensitivity analysis plot for VAP incidence.

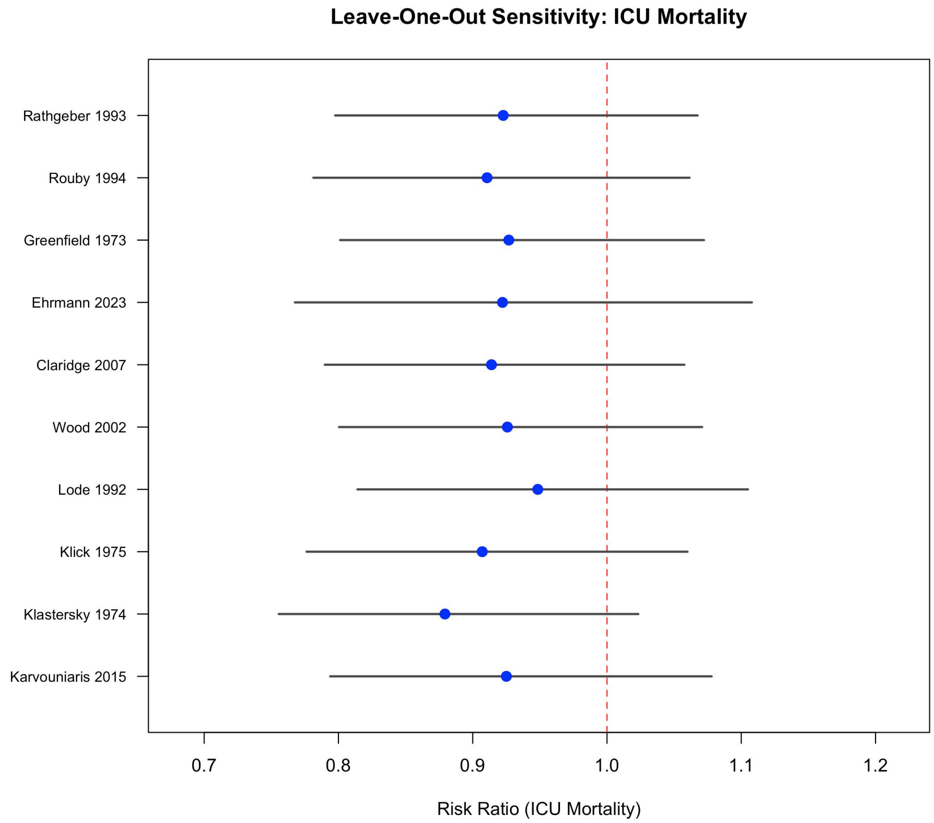

Figure S5. Leave-one-out sensitivity analysis plot for ICU mortality.
